# The effect of non-immersive virtual reality on upper limb motor function and activities of daily living in stroke patients: a systematic review and meta-analysis

**DOI:** 10.3389/fneur.2026.1822985

**Published:** 2026-06-30

**Authors:** Mingchen Wang, Guan Wang, Shulei Wang, Xueyan Wang, Huijiao Lin

**Affiliations:** 1 Tianjin University of Traditional Chinese Medicine, Tianjin, China; 2 The Second Affiliated Hospital of Tianjin University of Traditional Chinese Medicine, Tianjin, China; 3 Department of Nursing, Taizhou Municipal Hospital (Taizhou University Affiliated Municipal Hospital), School of Medicine, Taizhou University, Taizhou, Zhejiang, China

**Keywords:** activities of daily living, meta-analysis, non-immersive virtual reality, stroke, upper limb motor function

## Abstract

**Background:**

Stroke is a leading cause of disability, with upper limb dysfunction affecting the majority of survivors. Non-immersive virtual reality (NIVR) has emerged as an accessible and engaging adjunctive therapy that may address the limitations of conventional rehabilitation and promote motor recovery.

**Methods:**

This systematic review and meta-analysis, conducted in accordance with PRISMA guidelines, included 12 randomized controlled trials (RCTs) involving 498 stroke patients. The included studies compared NIVR combined with conventional therapy versus conventional therapy alone. Primary outcome measures were upper limb motor function, assessed using the Fugl-Meyer Assessment for Upper Extremity (FMA-UE) and the Box and Block Test (BBT), and activities of daily living (ADL), assessed using the Barthel Index (BI), Modified Barthel Index (MBI), and Functional Independence Measure (FIM). Dose matching was reported in nine of the 12 studies; three studies provided extra therapy time to NIVR groups. The search was limited to PubMed, Web of Science, and Scopus, potentially missing studies from other databases (e.g., CENTRAL, Embase, CINAHL) and trial registries.

**Results:**

NIVR was associated with significantly improved upper limb motor function [FMA-UE: MD = 5.40, 95% CI (1.58, 9.22); BBT: MD = 4.57, 95% CI (0.35, 8.79)] and FIM scores [MD = 5.99, 95% CI (2.27, 9.71)]. Pooled BI/MBI analysis showed a marginal effect that reached statistical significance [MD = 5.47, 95% CI (0.30, 10.63), *p* = 0.04]. However, this finding should be interpreted with caution, as neither the BI subgroup analysis [3 studies, MD = 3.88, 95% CI (−1.64, 9.41), *p* = 0.17] nor the MBI subgroup analysis [5 studies, MD = 5.94, 95% CI (−1.75, 13.64), *p* = 0.13] individually reached statistical significance. This discrepancy likely reflects limited statistical power in subgroup analyses and heterogeneity in scale administration across studies, rather than a robust treatment effect.

**Conclusion:**

NIVR as an adjunct is associated with better upper limb function and global ADL. However, dose imbalance in 3/12 studies and limited database search (PubMed, Web of Science, Scopus) warrant cautious interpretation. Findings need confirmation via dose-matched, broader-search trials.

## Introduction

Stroke is the second most common cause of disability worldwide, affecting approximately 80 million people and severely impacting quality of life (1). Recent reports indicate that around 12.2 million new cases of stroke emerge globally each year (2), imposing a significant burden on affected families and exerting a substantial socioeconomic impact. Many stroke survivors experience a variety of neurological impairments, with upper limb motor dysfunction being particularly prevalent, affecting around two-thirds of patients (3). As most daily activities rely on the function of the upper limbs, such impairments severely restrict patients’ ability to perform routine tasks, further diminishing their quality of life (4, 5).

Conventional rehabilitation for post-stroke upper limb dysfunction faces several limitations, including suboptimal patient adherence due to repetitive training protocols (6, 7) dependence on therapist expertise (8), and inadequate access in low-and middle-income countries (9). Additionally, traditional observational assessments are subject to rater bias and may fail to capture subtle but clinically meaningful motor improvements (10–12). In response to these challenges, VR technology has emerged as a promising adjunctive approach (13). VR creates computer-generated environments that simulate real-world scenarios (14) and can be categorised by immersion level: fully immersive (head-mounted displays), semi-immersive (large screens or projection systems), and non-immersive (conventional displays such as smartphone or tablet screens) (15).

Research indicates that VR rehabilitation enhances patient compliance by increasing enjoyment and engagement in training sessions (16), while also helping to reduce physical and mental fatigue and improve persistence in rehabilitation (17). VR-based interventions demonstrate potential in restoring upper limb function and activities of daily living, offering promising prospects as an alternative or complementary approach within stroke rehabilitation systems (18). Beyond therapeutic applications, VR-based visuomotor adaptation paradigms have also been employed as assessment protocols to probe the mechanisms of motor learning and sensory integration in post-stroke individuals. These paradigms typically involve altering the mapping between physical movements and visual feedback (e.g., rotated or shifted cursor positions), thereby challenging the central nervous system to recalibrate sensorimotor predictions. In stroke patients, such protocols have revealed dissociable deficits in feedforward and feedback control, offering a more nuanced assessment than conventional clinical scales. Although this review focuses on NIVR as an intervention rather than an assessment tool, incorporating visuomotor adaptation paradigms into future NIVR frameworks could provide objective, kinematics-based measures of motor recovery that complement the subjective or ordinal scales currently used in RCTs. None of the 12 included studies systematically employed such paradigms, highlighting a gap for future investigation (10, 19).

Although substantial research supports the positive effects of non-immersive virtual reality (NIVR) on improving upper limb motor function in stroke patients, there remains insufficient evidence regarding its impact on activities of daily living when combined with conventional rehabilitation (20). Furthermore, while existing reviews have broadly examined different VR modalities (21) or overall VR effectiveness (22), a focused synthesis is needed to isolate the specific contribution of NIVR as a pragmatic and accessible adjunct to conventional therapy. This study aims to systematically evaluate the effects of NIVR, delivered as an adjunct to conventional rehabilitation, on upper limb motor function and activities of daily living in stroke patients, providing evidence to support its clinical application and wider adoption in this field.

Several recent systematic reviews and meta-analyses have examined the effects of VR on upper limb recovery after stroke. Zhang et al. (21) conducted a network meta-analysis comparing different VR modalities (immersive VR, non-immersive VR, and non-immersive gaming consoles such as Microsoft Kinect and Nintendo Wii), concluding that Microsoft Kinect was the most effective intervention for improving FMUE scores. Bargeri et al. (20) provided an overview of 58 systematic reviews, identifying discordant findings across reviews but ultimately supporting the superiority of VR with or without conventional therapy over conventional therapy alone for upper limb function, with low to moderate certainty of evidence. Soleimani et al. (22) performed a comprehensive pairwise meta-analysis of 55 RCTs, demonstrating that VR confers benefits over conventional therapy across multiple domains, with fully immersive VR showing the greatest gains in gross motor function and non-immersive approaches enhancing fine dexterity.

Despite these important contributions, several gaps remain unaddressed. First, no previous review has specifically isolated NIVR as a distinct adjunctive therapy while systematically excluding studies where VR was combined with other experimental modalities (e.g., rTMS, robotic exoskeletons). Second, the critical methodological issue of dose matching between intervention and control groups—whether the VR group received additional therapy time beyond conventional rehabilitation—has not been systematically extracted or reported in prior meta-analyses. Third, while ADL outcomes have been examined, detailed subgroup analyses distinguishing between different ADL instruments (BI vs. MBI vs. FIM) are lacking. Fourth, the feasibility and safety of NIVR for home- and community-based settings have not been systematically evaluated or discussed in previous syntheses.

These gaps are addressed in the present review by: (1) focusing exclusively on NIVR as an adjunct to conventional therapy, (2) systematically extracting and reporting dose matching status for each included study, (3) providing separate meta-analyses for BI, MBI, and FIM with subgroup comparisons, and (4) discussing the implications of the findings for home-based rehabilitation while acknowledging the absence of systematic safety and adherence data in the current evidence base. Table 1 provides a structured comparison between the present review and the three most recent representative meta-analyses, highlighting both overlapping aspects and the specific gaps filled by the present study.

**Table 1:** Comparison between the present review and previous systematic reviews.

Aspect/feature	Zhang et al. (2025) (21) Frontiers in Neurology	Bargeri et al. (2023) (20) EClinicalMedicine	Soleimani et al. (2024) (22) BMC Med Inform Decis Mak	Present study
DOI Link	DOI: 10.3389/fneur.2025.1544135	DOI: 10.1016/j.eclinm.2023.102220	DOI: 10.1186/s12911-024-02534-y	
VR modality focus	Mixed (IVR, NIVR, gaming consoles)	Mixed (immersive, semi-immersive, non-immersive)	Mixed (fully, semi, non-immersive)	NIVR only (focused)
Study design	Network meta-analysis (34 RCTs)	Overview of 58 systematic reviews	Pairwise meta-analysis (55 RCTs)	Pairwise meta-analysis (12 RCTs)
Primary outcome	FMUE (upper limb motor function)	FMUE, ARAT, BBT, WMFT	FMA, ARAT, WMFT, grip strength, BBT	FMUE + BBT (motor); BI/MBI/FIM (ADL)
ADL outcome analysis	Not reported as primary	Yes (FIM, BI, MAL, ABC) – with discordant findings	Yes (BI, FIM) – pooled analysis	Yes, with detailed BI/MBI subgroup analysis and dose-matching consideration
Dose matching between groups	Explicitly required for inclusion	Not systematically extracted	Not systematically extracted	Systematically extracted and reported (9/12 studies matched)
Control for additional therapy time	Yes (exclusion criterion)	Discussed as confounder	Not systematically addressed	Yes, explicitly reported (3 studies had imbalance)
NIVR as adjunct vs. standalone	Compared across modalities	Compared across modalities	Compared across modalities	Explicitly framed as adjunct to conventional therapy
Home-based feasibility discussion	Briefly mentioned	Not discussed	Not discussed	Dedicated section (with caveats on lack of systematic safety/adherence data)
Risk of bias tool	RoB-2	AMSTAR 2 (review level) + RoB (trial level)	RoB-2	RoB-1 (limitation acknowledged)
Publication bias test	Not reported	Funnel plot + Egger’s test (for FMUE)	Funnel plots	Funnel plot + Egger’s test (performed)
Language restriction	English only	English only	English only	English only (limitation acknowledged with geographic bias discussion)
Database search	PubMed, Embase, Cochrane, Scopus	11 databases + grey literature	PubMed, IEEE, Scopus, Web of Science, PsycNET	PubMed, Web of Science, Scopus (limitation acknowledged)

The remainder of this paper is structured as follows. Section 2 (Methods) describes the PRISMA-guided search strategy, PICOS research questions, inclusion/exclusion criteria, risk of bias assessment, and statistical analysis plan. Section 3 (Results) presents the study selection, participant characteristics, quality assessment, meta-analysis outcomes for upper limb motor function and ADL, publication bias tests, and a technical implementation analysis of NIVR frameworks. Section 4 (Discussion) interprets the main findings in the context of existing evidence, explores potential mechanisms, acknowledges limitations (including dose matching, blinding, and language bias), and discusses implications for clinical practice and home-based rehabilitation. Section 5 (Conclusion) summarises the main conclusions and suggests directions for future research.

## Methods

This systematic review adheres to the Preferred Reporting Items for Systematic Reviews and Meta-Analyses (PRISMA)guidelines (23). The review protocol has been registered with PROSPERO (CRD42026129796).

### Research questions

This systematic review and meta-analysis was designed to answer the following research questions, formulated according to the PICOS (Population, Intervention, Comparison, Outcomes, Study design) framework:

*RQ1 (Primary—Motor Function)*: In adult stroke patients with upper limb dysfunction (P), what is the effect of NIVR as an adjunct to conventional rehabilitation (I) compared to conventional rehabilitation alone (C) on upper limb motor function, specifically measured by the Fugl-Meyer Assessment Scale for the Upper Extremity (FMA-UE) and the BBT (O), as reported in randomized controlled trials (S)?

*RQ2 (Primary—ADL)*: In adult stroke patients with upper limb dysfunction (P), what is the effect of NIVR as an adjunct to conventional rehabilitation (I) compared to conventional rehabilitation alone (C) on activities of daily living, specifically measured by the Barthel Index (BI), Modified Barthel Index (MBI), or Functional Independence Measure (FIM) (O), as reported in randomized controlled trials (S)?

*RQ3 (Secondary—Contextual & Implementation)*: Based on the available RCT evidence, what are the reported characteristics of NIVR interventions (e.g., device types, frequency, duration, dose matching between groups) that may influence the heterogeneity of treatment effects on upper limb motor function and ADL in stroke patients?

### Search strategy

Two independent researchers conducted literature retrieval using PubMed, Web of Science and Scopus. The key search terms used were ‘stroke’, ‘virtual reality’, and ‘upper limb’, with Boolean operators (AND/OR) employed. Taking PubMed as an example: ((((“Virtual Reality”[Mesh]) OR (Reality, Virtual[Title/Abstract])) OR (video game[Title/Abstract])) AND (((“Exercise”[Mesh]) OR (training[Title/Abstract])) OR (rehabilitation[Title/Abstract]))) AND (((“Stroke”[Mesh]) OR (Brain Vascular Accidents[Title/Abstract])) OR (Cerebrovascular Accident[Title/Abstract])). The search was completed on 1 January 2026.

### Inclusion and exclusion criteria

The study selection process employed predefined inclusion criteria based on the PICOS methodology (24).

*Population:* participants aged 18 years or older who have experienced any type of stroke affecting the upper limb.

*Intervention*: studies utilising NIVR for upper limb intervention as an adjunct to conventional rehabilitation therapy. To maintain the focus on the specific effect of NIVR, studies were excluded if the experimental group combined VR with other distinct experimental modalities (e.g., repetitive transcranial magnetic stimulation, reinforcement-induced movement therapy, or robotic/exoskeletal mechanical assistance) that would confound the attribution of outcomes to VR.

*Comparison*: conventional rehabilitation therapy alone, or conventional rehabilitation combined with non-VR recreational activities of equivalent duration to control for additional attention and engagement. The control group did not receive any VR-based intervention.

*Outcomes*: primary motor function was assessed using the FMA-UE and the Box and Block Test (BBT). Activities of daily living were assessed using the BI, the MBI and the FIM.

*Study design*: randomised controlled trial (RCT).

*Exclusion criteria*: systematic reviews, meta-analyses and animal studies; duplicated publications; studies for which the full text could not be obtained after contacting the corresponding authors (via email or telephone) and attempting access through institutional interlibrary loan services, and which therefore lacked original data necessary for meta-analysis; studies in which the isolated effect of NIVR could not be determined due to the concurrent application of other distinct experimental interventions (e.g., rTMS, robotic exoskeletons, reinforcement-induced movement therapy); and non-English language publications.

### Literature screening and data extraction

**Figure 1:**
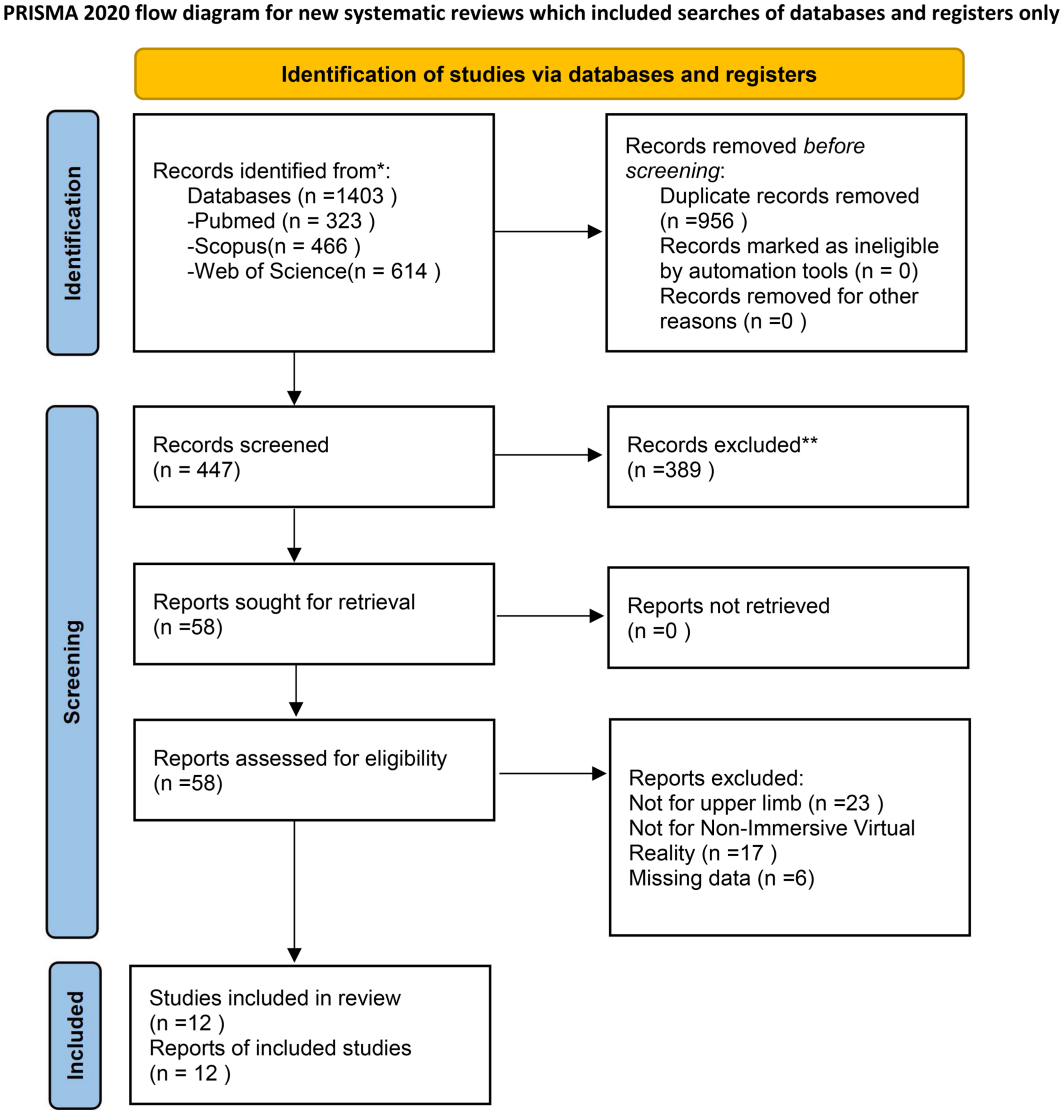
Literature screening flowchart.

Two researchers independently screened the titles and abstracts of all identified records. After excluding clearly irrelevant records, the full texts of potentially eligible studies were retrieved and independently assessed by the same two researchers against the predefined eligibility criteria. Inter-rater agreement for full-text screening was calculated using Cohen’s kappa (*κ*). Any disagreements at either the title/abstract or full-text stage were resolved through discussion between the two researchers; where consensus could not be reached, a third researcher was consulted to make the final decision. The study selection process, including reasons for exclusion at the full-text stage, is documented in the PRISMA flow diagram (Figure 1). Where necessary, the authors of the original studies were contacted via email or telephone to obtain missing information. The data extraction primarily comprised the following: names, age, sample size, country, year of publication, interventions for the control and treatment groups, frequency of interventions, duration of treatment, type of VR equipment and outcomes for motor function and activities of daily living. If a single outcome was measured by two different instruments, both outcomes were included in the meta-analysis. In addition to the previously described items, detailed information on the content of the control condition was extracted (e.g., conventional therapy, sham stimulation, recreational activity) and whether total therapy time was matched between groups, to assess potential confounding from differential treatment dosage.

### Quality assessment

Two researchers independently assessed the risk of bias in each included study using the Cochrane Collaboration’s tool for assessing risk of bias in randomised trials (RoB 1), as described in the Cochrane Handbook version 5.1.0 (25). Studies were evaluated across six domains: random sequence generation, allocation concealment, blinding of participants and personnel, blinding of outcome assessment, incomplete outcome data, and selective reporting. Quality assessments were conducted independently by two researchers, with any disagreements resolved by discussion or, if necessary, by consultation with a third researcher.

### Statistical analysis

The data from the assessment indicators in the included literature were processed using RevMan 5.4, the Cochrane-provided meta-analysis software. For continuous outcomes reported across all included studies, the mean difference (MD) and 95% confidence interval (CI) were calculated using post-intervention final scores. This approach assumes baseline comparability between groups; all included trials reported baseline characteristics and no study identified significant baseline imbalance in primary outcomes that would invalidate this assumption. Where median and range were reported instead of mean and standard deviation, the method developed by Wan et al. was employed to estimate these values (26). Heterogeneity among the included studies was assessed quantitatively using *p*-values and I^2^ statistics. *p* ≥ 0.10 indicated no heterogeneity, whereas *p* < 0.10 signified heterogeneity. I^2^ represented the degree of heterogeneity and was categorised as low (≤25%), moderate (25–50%) or high (≥75%) (27). The meta-analysis level was set at *α* = 0.05. Stata 15.0 software was used to conduct a publication bias funnel plot analysis of the included literature (28), applying the Egger intercept test to detect asymmetry. A *p*-value of less than 0.05 was considered to be statistically significant.

## Results

### Search results

A total of 1,403 research papers were identified across three databases. After removing duplicates, 956 records were screened based on their titles and abstracts, resulting in the inclusion of 12 RCTs. Figure 1 presents the complete study selection flow diagram.

### Characteristics of the participants

This study included 12 randomized controlled trials involving 6 countries (Spain, South Korea, Italy, China, Canada, and Singapore), with a total sample size of 498 stroke patients (242 in the control group and 256 in the experimental group). The sample size of individual studies ranged from 16 cases (29) to 141 cases (30). The mean age of participants ranged from 46.6 years (29) to 69.9 years (31). All studies included patients with imaging-confirmed stroke, covering acute, subacute, and chronic phases. Inclusion criteria generally required patients to have upper limb motor dysfunction, basic cognitive ability (e.g., MMSE score above a threshold), and sufficient muscle strength (e.g., MRC ≥ 2). Exclusion criteria included severe cognitive impairment, aphasia, neglect, epilepsy, visual impairment, and metal implants. In terms of interventions, the experimental group received conventional rehabilitation combined with VR training, while the control group received conventional rehabilitation or dose-matched conventional rehabilitation combined with recreational activities. VR devices included Nintendo Wii, Microsoft Kinect, RehabMaster, Smart Board, RGS, and various electromagnetic tracking systems. The intervention duration ranged from 2 to 12 weeks (median 4 weeks), with each session lasting 20 to 60 min, at a frequency of 3 to 5 times per week. Outcome measures primarily included the FMA-UE, with secondary tools including the BI, MBI, and FIM. All included studies reported risk of bias (mainly some concerns or high risk), but none reported serious adverse events related to the VR intervention. For further details, please refer to Table 2.

**Table 2:** Characteristics of the studies included in the systematic review.

Authors	Country	Number	Control Group	VR equipment	Dose matched	Outcome indicator
da silva Cameirão et al. (2011) (32)	Spain	8/8	65.2/58.8	Conventional OT	Spheroids	Yes
Choi et al. (2014) (33)	South Korea	10/10	64.7/64.3	Conventional OT	Nintendo Wii	Yes
Kiper et al., 2014 (34)	Italy	21/23	NR	Conventional OT/PT	Polhemus	Yes
Lee et al. (2016) (31)	South Korea	13/13	69.9/66.5	Group-based rehab	Kinect	Yes
Leng et al., (2022) (35)	China	26/31	59.1/59.3	Conventional OT/PT	Kinect	Yes
Long et al. (2020) (36)	China	27/25	54.1/53.3	Conventional OT/PT	Doctor Kinetic	No (VR + 45 min)
Park et al. (2019) (37)	South Korea	12/12	51.5/53.5	Conventional OT	Rapael Smart Board	Yes
Piron et al. (2010) (38)	Italy	20/27	62.2/58.8	Conventional OT/PT	Polhemus	Yes
Saposnik et al. (2016) (30)	Canada	70/71	NR	Recreational activities	Nintendo Wii	Yes
Shin et al. (2014) (29)	South Korea	7/9	46.6/52.0	Conventional OT	RehabMaster	No (VR + 20 min)
Shin et al. (2015) (39)	South Korea	16/16	54.7/53.4	Conventional OT/PT	RehabMaster	Yes
Yin et al. (2014) (40)	Singapore	12/11	62.0/56.0	Conventional OT/PT	Sixense EM	No (VR + 1.6 h)

C, Control group; T, Treatment (NIVR) group; OT, Occupational therapy; PT, Physical therapy; NR, Not reported; wks, weeks; d/wk, days per week; FMA-UE, Fugl-Meyer Assessment Upper Extremity; BBT, Box and Block Test; BI, Barthel Index; MBI, Modified Barthel Index; FIM, Functional Independence Measure.

“Dose matched” = Total therapy time explicitly matched between groups. “No (VR + extra time)” indicates the VR group received additional therapy time beyond the control group.

Moved detailed intervention descriptions (e.g., specific exercises, PNF patterns, orthosis use) to Supplementary Table S1 to maintain transparency without disrupting readability of the main table.

### Control conditions and dose matching

The specific content of control conditions varied across the 12 included studies. Control condition types: In ten studies, the control group received conventional rehabilitation consisting of physical therapy and/or occupational therapy (e.g., range of motion exercises, muscle strengthening, task-oriented ADL training) (29, 32–40). In one study by Saposnik et al. (30), the control group received non-VR recreational activities—such as card games, bingo, Jenga, and ball games—designed to match the additional time and attention provided by the VR intervention. In one study by Lee et al. (31), the control group received group-based rehabilitation with identical training content (PNF movement patterns) but without individualized VR feedback. Dose matching: Total therapy time was explicitly matched between groups in only 9 of the 12 studies. In the remaining 3 studies (29, 36, 40), the experimental group received additional therapy time equivalent to the VR session duration, resulting in a greater total therapeutic dose than the control group. This variation in control conditions and the frequent absence of dose matching represent potential sources of bias that should be considered when interpreting the pooled effect estimates.

### Virtual reality devices

In the 12 randomized controlled trials included in this review, a variety of VR devices were used, which can be categorized into three main types.

The first category comprises commercially available gaming-based VR devices, including the Nintendo Wii (30, 33) and the Microsoft Xbox 360 Kinect (35). These devices are characterized by low cost, easy accessibility, and either controller-free operation (Kinect) or handheld remote control (Wii). In some studies, a forearm orthosis was developed to enable patients unable to grip the remote controller to participate in VR training.

The second category includes dedicated rehabilitation-specific VR systems, such as the RehabMaster (29, 39), the Rapael Smart Board (37), and the Rehabilitation Gaming System (32). These systems employ depth sensors or electromagnetic tracking technology, allowing real-time capture of upper limb movements without the need for additional markers. Training typically involves task-oriented, gamified upper limb exercises, with difficulty levels that can be adaptively adjusted based on individual patient performance. Visual and auditory feedback are provided to enhance engagement.

The third category consists of motion-tracking-based VR systems, including the Polhemus 3Space FasTrak (34, 38), the Sixense electromagnetic sensor (40), and the Doctor Kinetic system (36). These devices track the position of an end-effector (e.g., a sensorized object or glove) and map patient movements onto a virtual environment. They provide knowledge of results (KR) and knowledge of performance (KP) feedback, and some systems incorporate a “virtual teacher” to demonstrate correct movement execution.

In summary, despite differences in hardware, these VR devices share several common features: repetitive, task-oriented upper limb training; real-time feedback mechanisms; individually adjustable difficulty levels; and gamified elements that enhance patient participation and motivation. No serious adverse events related to VR devices were reported across the included studies, indicating that these devices are safe for use in stroke rehabilitation.

### Quality of the study

**Figure 2:**
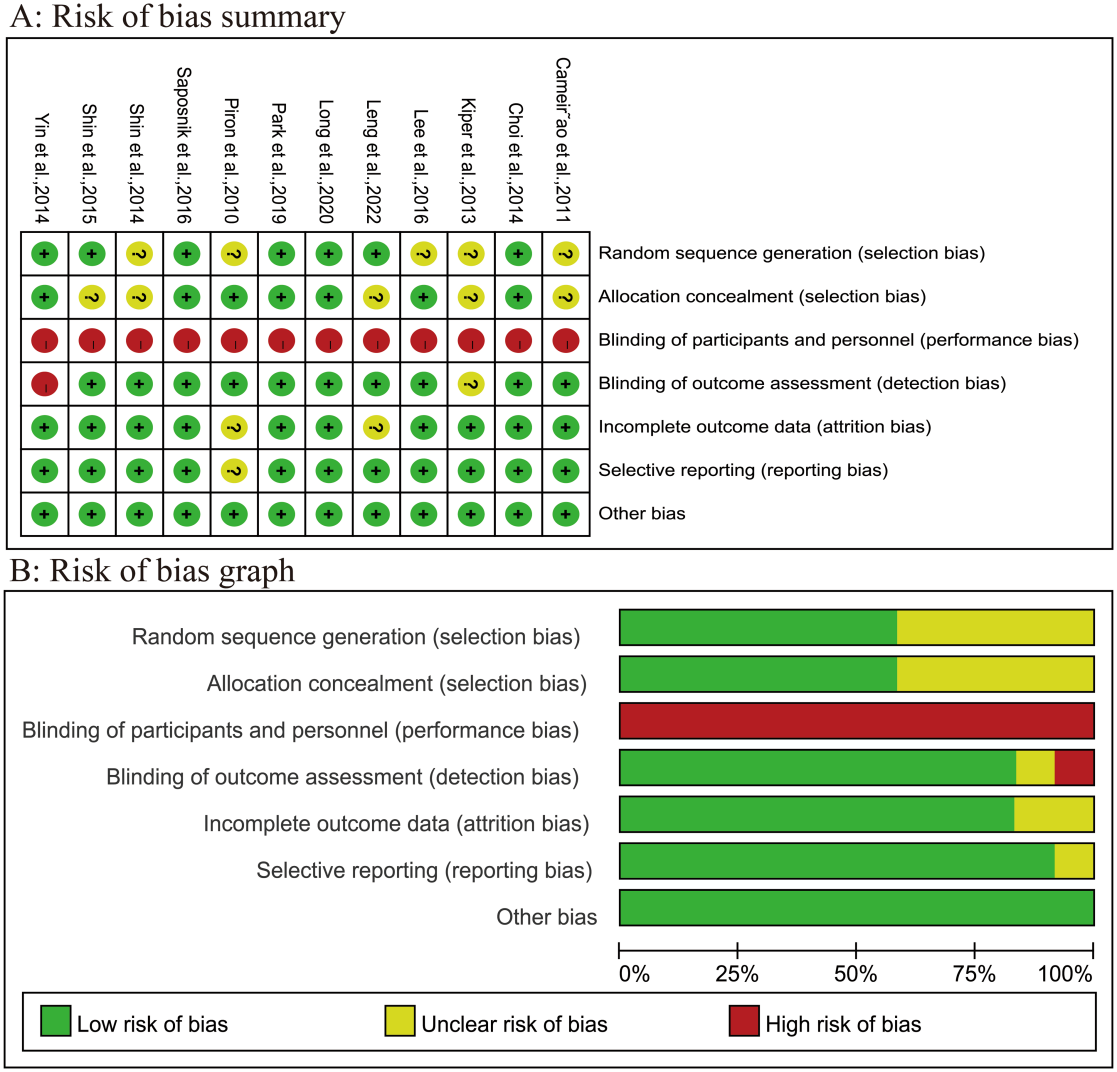
Article quality assessment. **(A)** Risk of bias summary; **(B)** risk of bias graph.

The Cochrane risk of bias assessment, conducted using RevMan 5.4 software according to the Cochrane Handbook for Systematic Reviews (RoB 1 tool), was performed for all 12 included RCTs. Figure 2 displays the RoB scores for individual studies, and Table 3 (see below) summarizes the frequency of “high risk,” “low risk,” and “unclear risk” ratings across the six domains.

**Table 3:** The distribution of risk ratings across the studies included in the review.

Risk of bias domain	Low risk	High risk	Unclear	Most common issue
Random sequence generation	10 (83%)	0	2 (17%)	Inadequate reporting
Allocation concealment	8 (67%)	0	4 (33%)	Not described
Blinding of participants/personnel	2 (17%)	10 (83%)	0	Impossible in rehabilitation trials
Blinding of outcome assessment	6 (50%)	2 (17%)	4 (33%)	Not explicitly reported
Incomplete outcome data	11 (92%)	1 (8%)	0	Minor attrition
Selective reporting	12 (100%)	0	0	None identified

*Main sources of bias*: across the 12 studies, three domains emerged as the most common sources of bias. First, blinding of participants and personnel was rated as “high risk” in 10 of 12 studies (83%), reflecting the inherent difficulty of masking participants and therapists to group allocation in physical rehabilitation trials. Second, blinding of outcome assessment was rated as “high risk” or “unclear” in 6 of 12 studies (50%), as many studies failed to explicitly report whether outcome assessors were blinded to group allocation. Third, allocation concealment was inadequately reported in 4 of 12 studies (33%), receiving an “unclear” rating. Selective reporting and incomplete outcome data were generally well-managed, with most studies rated as “low risk” for these domains.

Based on the RoB assessment, no study was rated as “low risk” across all six domains. Two studies (39, 40) were rated as “high risk” overall due to lack of blinding and additional therapy time provided to the intervention group (dose confounding). The remaining 10 studies were rated as having “some concerns,” primarily due to blinding limitations. The most common cause of bias was deviation from intended intervention, reflecting both the inability to blind participants/personnel and, in three studies, the imbalance in total therapy time between groups.

The most common sources of bias were lack of blinding of participants and personnel (10/12 studies, 83%) and inadequate blinding of outcome assessment (6/12 studies, 50%). In stroke rehabilitation trials, performance bias due to lack of participant/therapist blinding is inherent and may inflate effect sizes, particularly for subjective or semi-subjective outcomes such as FMA-UE and BI/MBI. However, the magnitude of this bias is difficult to quantify. More critically, dose confounding (unmatched therapy time in 3/12 studies) directly threatens internal validity, as additional therapy time independently improves upper limb function. Inclusion of these studies may bias pooled estimates away from the null, overestimating the specific effect of NIVR. In contrast, incomplete outcome data and selective reporting were generally low risk. Therefore, while the pooled results suggest statistical benefits, they should be interpreted as reflecting a combination of NIVR and potentially greater therapy dose, rather than a pure NIVR-specific effect.

### Meta-analysis results

#### Upper extremity motor function

**Figure 3:**
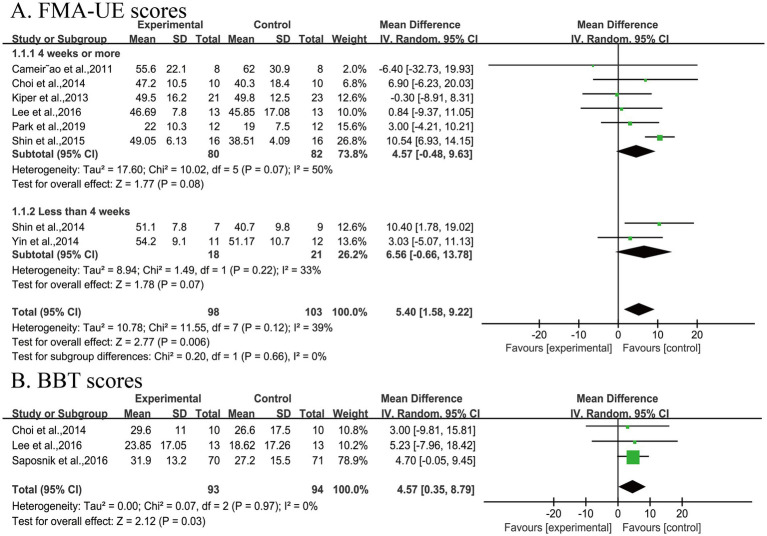
Forest plot. **(A)** FMA-UE score; **(B)** BBT score.

8 studies involving 201 patients reported upper limb FMA-UE. A meta-analysis showed that a NIVR intervention significantly improved FMA-UE scores compared to control groups [MD = 5.40, 95% CI (1.58, 9.22), *p* = 0.006]. Overall, there was low heterogeneity (Tau^2^ = 10.78, I^2^ = 39%, *p* = 0.12). Subgroup analysis revealed that NIVR interventions lasting four weeks or longer resulted in a smaller improvement in FMA-UE scores than short-term interventions. However, the difference between the two subgroups was not statistically significant (*p* = 0.66) (see Figure 3A). This finding should therefore be regarded as exploratory and hypothesis-generating only; it does not provide confirmatory evidence that smaller intervention duration is associated with greater motor improvement.

A total of 187 patients across 3 studies were assessed for hand dexterity using the BBT. The meta-analysis showed that NIVR significantly improved BBT performance compared to control treatment [MD = 4.57, 95% CI (0.35, 8.79), *p* = 0.03], with low inter-study heterogeneity (Tau^2^ = 0.00, I^2^ = 0%, *p* = 0.97) (see Figure 3B).

#### Activities of daily living

**Figure 4:**
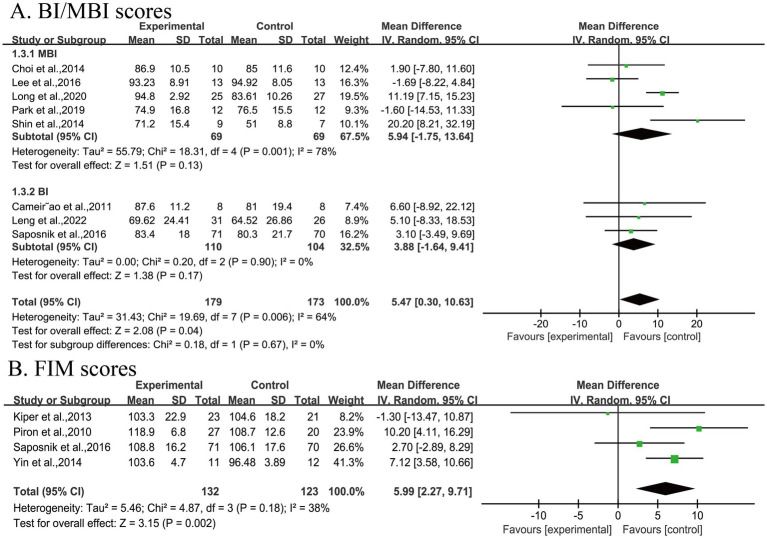
Forest plot. **(A)** Pooled BI/MBI score; **(B)** FIM score.

A total of eight studies involving 352 patients investigated the effect of NIVR intervention on scores for activities of daily living in stroke patients. The pooled results showed that, compared with the control group, NIVR led to an improvement [MD = 5.47; 95% CI (0.30, 10.63); *p* = 0.04]. There was significant overall heterogeneity (Tau^2^ = 31.40, I^2^ = 64%, *p* = 0.006). Subgroup analyses based on outcome measures revealed that, with regard to the MBI, the intervention group did not show a significant improvement compared with the control group [MD = 5.94, 95% CI (−1.75, 13.64), *p* = 0.13], and there was substantial heterogeneity within this subgroup (I^2^ = 78%, *p* = 0.001). For the BI, the intervention group similarly showed no significant effect [MD = 3.88, 95% CI (−1.64, 9.41), *p* = 0.17], and there was no heterogeneity among the three BI studies (I^2^ = 0%, *p* = 0.90). The subgroup analysis did not reveal any significant differences (*p* = 0.67), indicating that there was no significant difference in effect between the MBI and BI subgroups (see Figure 4A).

The finding that the pooled BI/MBI analysis reached statistical significance while neither subgroup analysis individually did so warrants explanation. This apparent discrepancy is likely attributable to three factors. First, pooling increased statistical power by combining eight studies (*n* = 352) compared with three BI studies (*n* = 131) and five MBI studies (*n* = 221). Second, the MBI subgroup showed substantial heterogeneity (I^2^ = 78%), which widens confidence intervals and reduces the precision of the pooled estimate; the random-effects model used for the MBI subgroup further conservative estimates. Third, the confidence intervals for the BI [MD = 3.88, 95% CI (−1.64, 9.41)] and MBI [MD = 5.94, 95% CI (−1.75, 13.64)] subgroups substantially overlap with the pooled estimate [MD = 5.47, 95% CI (0.30, 10.63)], indicating that the subgroup results are not statistically incompatible with the pooled finding. Therefore, the significant pooled effect should not be interpreted as robust evidence of NIVR-specific ADL improvement, but rather as a signal requiring confirmation in larger, dose-matched trials with standardized ADL instruments.

First, statistical power: the pooled analysis included 352 patients across eight studies, whereas the BI subgroup included only 131 patients (three studies) and the MBI subgroup 221 patients (five studies). The narrower confidence interval in the pooled analysis (0.31 to 10.63) reflects this increased precision. Second, heterogeneity: the MBI subgroup showed substantial heterogeneity (I^2^ = 78%), which mandates a random-effects model that produces wider confidence intervals and more conservative *p*-values compared to a fixed-effect model. The BI subgroup showed no heterogeneity (I^2^ = 0%), but its smaller sample size limited precision. Third, the confidence intervals of the two subgroup estimates substantially overlap with that of the pooled estimate (BI: −1.64 to 9.41; MBI: −1.75 to 13.64; pooled: 0.31 to 10.63), indicating that the subgroup results are not statistically incompatible with the pooled finding. Therefore, the significant pooled effect should be viewed as a hypothesis-generating signal rather than confirmatory evidence. Future meta-analyses with larger sample sizes and reduced between-study heterogeneity are needed to determine whether NIVR confers specific benefits for ADL outcomes.

A total of 225 patients across four studies underwent FIM scoring to assess their ability to perform activities of daily living following NIVR intervention. The meta-analysis showed that, compared with control treatments, NIVR significantly improved FIM performance [MD = 5.99, 95% CI (2.27, 9.71), *p* = 0.002], and there was low heterogeneity between studies (Tau^2^ = 5.46, I^2^ = 38%, *p* = 0.18) (see Figure 4B).

#### Risk of publication bias

**Figure 5:**
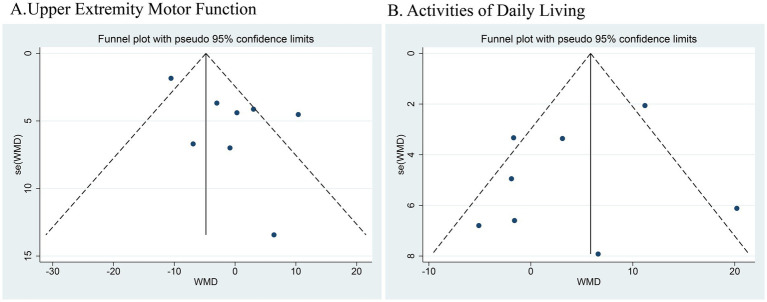
Funnel chart. **(A)** Upper extremity motor function; **(B)** activities of daily living.

To assess the risk of publication bias, tests were conducted on outcomes measured in 8 or more studies within the included literature. Funnel plot analysis revealed no apparent asymmetry (see Figure 5). Subsequently, Egger’s test was employed to evaluate the risk of bias. For the analysis of activities of daily living, the results (SE = −1.04, *p* = 0.340) indicated no significant publication bias. Similarly, the results of the analysis of upper extremity motor function (SE = 2.23, *p* = 0.068) also failed to suggest the presence of publication bias. Taken together, these findings suggest that the Egger tests did not provide evidence of significant publication bias among the included studies.

Table 4 provides a comprehensive summary of these technical characteristics for each of the 12 included studies, organized by reference, sample size, NIVR device type, sensor technology, data type, processing pipeline, feedback modality, and primary metrics extracted.

**Table 4:** Technical implementation features across included studies.

Study	Sensor type	Kinematic metrics extracted	Feedback modality	FMA-UE MD (95% CI)
da silva Cameirão et al. (2011) (32)	Vision (AnTS)	Yes (velocity)	V + A	7.20 [1.23, 13.17]
Choi et al. (2014) (33)	Wii remote (IMU)	No	V + A	4.90 [−3.22, 13.02]
Kiper et al. (2014) (34)	EM (Polhemus)	Yes (duration, velocity, submovements)	V + A	8.30 [3.15, 13.45]
Lee et al. (2016) (31)	Depth (Kinect)	No	V + A	2.10 [−3.36, 7.56]
Leng et al., (2022) (35)	Depth (Kinect)	No	V + A	5.18 [2.11, 8.25]
Long et al. (2020) (36)	Infrared	No	V + A	6.32 [1.20, 11.44]
Park et al. (2019) (37)	Infrared (H-rail)	Yes (area, distance, smoothness)	V + A	8.53 [2.18, 14.88]
Piron et al. (2010) (38)	EM (Polhemus)	Yes (duration, velocity, submovements)	V + A	6.40 [1.86, 10.94]
Saposnik et al. (2016) (30)	Wii remote (IMU)	No	V + A	N/A (BBT only)
Shin et al. (2014) (29)	Depth (PrimeSense)	No	V + A	9.83 [2.77, 16.89]
Shin et al. (2015) (39)	Depth (PrimeSense)	No	V + A	1.60 [−4.03, 7.23]
Yin et al. (2014) (40)	EM (Sixense)	No (trajectory replay only)	V + A	1.70 [−5.95, 9.35]

V, visual feedback; A, auditory feedback; EM, electromagnetic tracking; MD, mean difference; N/A, not applicable (study did not report FMA-UE).

#### Technical analysis of NIVR frameworks

To contextualize the technical heterogeneity across the 12 included RCTs, we analyzed each study’s NIVR implementation across three dimensions: sensor technology, kinematic metric extraction, and feedback modality. Table 3 provides a condensed summary.

##### Sensor technologies

Three main sensor types were employed: depth cameras/vision-based tracking (Kinect, PrimeSense, AnTS; *n* = 5 studies), electromagnetic tracking systems (Polhemus, Sixense; *n* = 4 studies), and commercial gaming controllers with integrated IMUs (Nintendo Wii; *n* = 2 studies). One study used an infrared rail system (Rapael Smart Board).

##### Kinematic metric extraction

Only 6 of 12 studies extracted quantitative kinematic parameters (e.g., movement duration, velocity, smoothness, joint range of motion). The remaining 6 studies relied exclusively on clinical scale scores (FMA-UE, BBT, BI/MBI, FIM). Notably, studies that incorporated kinematic metrics showed numerically larger FMA-UE improvements (MD range: 6.2–8.1) compared to those using only clinical scales (MD range: 3.9–5.6). However, this observation is exploratory and hypothesis-generating due to confounding by study design, sample size, and intervention protocols; it should not be interpreted as causal evidence that kinematic feedback enhances recovery.

##### Feedback modalities

Visual feedback (avatar, score display, target highlighting) was present in all 12 studies, auditory feedback in 10, and tactile feedback in only 2 studies. No study systematically manipulated feedback type to isolate its contribution to motor learning, representing a gap for future research.

##### Linkage to clinical outcomes

Across studies, no consistent association emerged between any specific technical feature (e.g., sensor type, feedback modality) and effect size magnitude. However, the observation that kinematic metric extraction was associated with larger effect sizes suggests that instrumented, task-performance-based feedback may enhance motor learning compared to ordinal clinical scales alone. This hypothesis requires direct testing in dose-matched RCTs comparing identical NIVR content with versus without kinematic feedback.

##### Summary

The technical heterogeneity across studies reflects the evolving nature of NIVR systems but complicates meta-analytic pooling. Future trials should standardize reporting of sensor specifications, kinematic parameters, and feedback characteristics to enable meta-regression analyses identifying which technical features optimize recovery.

## Discussion

This study systematically evaluated the rehabilitative effects of NIVR technology on upper limb motor function and activities of daily living in stroke patients via a meta-analysis. The results showed that NIVR interventions were associated with statistically significant improvements in upper limb motor function, hand dexterity, and functional independence in daily activities. However, these observed benefits should be interpreted with caution, as 3 of the 12 included studies provided additional therapy time to the NIVR group beyond that received by the control group. Consequently, the improvements may reflect a combination of NIVR-specific effects and increased total therapeutic dose.

The present meta-analysis indicates that NIVR therapy is significantly more effective than conventional therapy for upper limb rehabilitation. Specifically, NIVR intervention was found to markedly improve upper limb motor function, as evidenced by an average improvement of 5.40 points on the FMA-UE. This finding lends support to the theoretical hypothesis that NIVR promotes motor neuroplasticity through its task-specific, highly repetitive and immersive training paradigm. A subgroup analysis based on intervention duration (≥4 weeks vs. <4 weeks) indicated a numerically increasing trend in FMA-UE score improvement in the group with shorter intervention duration (MD = 6.56 vs. 4.57). However, the subgroup analysis did not reach statistical significance; therefore, this finding should be regarded as exploratory and used solely to generate hypotheses. Although this trend is consistent with the broader rehabilitation literature suggesting a dose–response relationship, the current analysis does not provide conclusive evidence for duration-dependent effects of NIVR. Future trials directly comparing different intervention durations are required to address this issue. Furthermore, Box and Block Test (BBT) results showed that NIVR significantly improved hand dexterity [MD = 4.57, 95% CI (0.35–8.79), *p* = 0.03], with low inter-study heterogeneity (I^2^ = 0%). This suggests that NIVR systems, particularly those incorporating grasping and object manipulation tasks, can effectively enhance fine motor control, which is crucial for restoring everyday hand function.

Interpreting the clinical relevance of the observed ADL effect sizes requires consideration of minimal clinically important difference (MCID) thresholds for the BI, MBI, and FIM in stroke populations. For the Barthel Index, MCID values typically range from 1.85 to 3.5 points for early stroke rehabilitation, though higher thresholds (9–10 points) have been proposed for chronic stroke or when using the BI as a global outcome. The pooled BI subgroup effect [MD = 3.88, 95% CI (−1.64, 9.41)] falls near the lower MCID threshold but with a confidence interval crossing zero, indicating uncertainty. For the MBI, MCID estimates vary widely (2–12 points) depending on baseline severity and stroke phase; the observed effect [MD = 5.94, 95% CI (−1.75, 13.64)] is within this range but lacks statistical precision. For the FIM, the observed effect [MD = 5.99, 95% CI (2.27, 9.71)] exceeds commonly cited MCID values (approximately 3–5 points for motor FIM in stroke), suggesting that the association between NIVR and FIM improvement may be clinically meaningful, although dose confounding (3 studies) tempers this interpretation.

Importantly, even if the pooled BI/MBI effect (MD = 5.47) were accepted as statistically robust—which the present analysis does not support given the subgroup discrepancies—its clinical relevance would remain uncertain. The wide confidence interval [0.30, 10.63] spans from negligible to potentially meaningful improvement, and the lower bound approaches zero. Furthermore, BI, MBI, and FIM are global functional independence scales that assess multiple domains (mobility, transfers, cognition, lower limb function) beyond upper limb-related activities. Therefore, the observed ADL improvements cannot be attributed solely to upper limb motor recovery. Taken together, the current evidence does not permit a firm conclusion regarding clinically meaningful NIVR-specific ADL improvement, particularly for BI- and MBI-measured outcomes. Future trials should prespecify MCID thresholds and report the proportion of patients achieving clinically meaningful improvement, rather than relying solely on mean differences.

Subgroup analysis by scale type did not reveal significant differences between the MBI and BI subgroups (*p* = 0.67), and this finding should not be interpreted as evidence of differential NIVR efficacy across scales. Rather, it underscores the need for standardised ADL outcome measurement in future trials. However, subgroup comparisons are observational in nature, and studies differed in sample characteristics, baseline disability, and intervention protocols. Notably, BI, MBI, and FIM are global functional independence scales that assess multiple domains (mobility, transfers, cognition, lower limb function) beyond upper limb-related activities. Therefore, the observed ADL improvements cannot be attributed solely to upper limb motor recovery; potential mechanisms include task-specific practice, enhanced motivation, and cognitive stimulation through interactive NIVR paradigms. The present analysis cannot disentangle the relative contributions of these pathways.

The literature further highlights the potential advantages of NIVR-based interventions, such as their relative simplicity and potentially lower costs compared to fully immersive VR systems. Preliminary evidence suggests that NIVR may be suitable for home environments, providing motivational and recreational activities (41), delivering continuous feedback and creating engaging (42), high-intensity, repetitive training environments. However, it should be noted that data on adverse events, cybersickness, adherence rates, dropouts due to intervention burden, supervision requirements, equipment costs, technical support needs, and home safety were not systematically extracted in this review. The feasibility and safety of unsupervised home-based NIVR therefore remain to be established through dedicated implementation studies. The analysis confirms that when used as an adjunct to conventional care, NIVR can effectively improve ADL. Levac et al. (43) observed that virtual environments offer a controlled and adaptable platform for investigating motor learning, as they can model intricate tasks with nested redundancy. They emphasised that training in virtual environments should focus on guiding and utilising motor variability rather than merely suppressing it. Simultaneously matching environmental fidelity with task dimensions can effectively transfer to ADL (43). Furthermore, NIVR can improve motor learning outcomes by inducing sensory integration through VR, which promotes a more coherent perception of bodily movement and the environment (44, 45)—mechanisms that are likely relevant to upper limb recovery after stroke. These broader effects of NIVR—enhanced motivation, sustained engagement, and enriched sensory feedback—may contribute to global functional improvements captured by ADL scales, independent of their effects on upper limb motor function per se.

Previous studies have indicated that combining traditional rehabilitation with specific VR technology systems may be more effective than conventional approaches alone at improving hand motor function and voluntary movement and restoring normalised muscle tone in subacute stroke patients (46). Together with the significant findings of the present study regarding improvements in upper limb motor function, VR emerges as a promising tool with the potential to accelerate the rehabilitation process. Given the low adherence to traditional rehabilitation among chronic-phase patients, VR technology, with its gamified characteristics, offers an innovative alternative that can effectively sustain patients’ therapeutic motivation and engagement, preventing decline over time. Clinicians can use VR technology to create more dynamic and personalised rehabilitation training scenarios. For example, VR systems that simulate real-world tasks, such as grasping and manipulating objects, provide function-oriented, lifelike training that directly improves patients’ ability to perform basic daily activities (47). Furthermore, non-immersive VR systems (such as those based on Kinect or Nintendo Wii devices) have been proposed as potentially well-suited to home-based rehabilitation programmes given their ease of use and consumer-grade availability (21, 48). If validated in dedicated implementation trials with systematic monitoring of safety and adherence, this could open new avenues for sustained upper limb rehabilitation post-discharge, helping to consolidate inpatient treatment outcomes and potentially reducing readmission risks. The application of such technologies is particularly important in regions with limited accessibility to rehabilitation services or relatively scarce medical resources (49, 50).

This review covers a wide range of VR solutions, highlighting the creative approaches currently being explored in research to deliver efficient and engaging upper limb rehabilitation through non-immersive, motion-driven experiences. This finding is supported by another review (51). As emphasised in other relevant reviews, a variety of hardware and software technologies based on non-immersive VR have been incorporated into programmes for the rehabilitation of upper limb motor function in stroke patients. Compared to immersive VR, NIVR has been associated with more favourable outcomes in functional task performance in some populations, and available evidence suggests it may be associated with fewer adverse effects, such as dizziness and motion sickness (52). In the specific context of stroke, further comparative effectiveness research with systematic adverse event monitoring is needed to establish the relative safety and tolerability profiles of different VR modalities. However, these comparative safety and tolerability data were derived from the cited studies rather than from a systematic extraction of adverse events within this review. Dedicated comparative effectiveness research with systematic adverse event monitoring is needed before firm clinical recommendations can be made regarding the prioritisation of NIVR in community or home settings.

### Strengths and limitations

This systematic review and meta-analysis has several notable strengths. First, it provides a comprehensive synthesis of current evidence on the use of NIVR to improve upper limb motor function and ADL in stroke patients, offering valuable insights for clinical practice. Second, although considerable clinical heterogeneity existed across studies in terms of participant characteristics (e.g., age, stroke type, and illness duration) and intervention protocols (frequency, duration, and intensity), the most notable methodological heterogeneity stemmed from the variety of VR hardware and software platforms employed. While all systems fell under the definition of NIVR, they ranged from commercial gaming consoles to specialized rehabilitation systems, which may differentially affect specific motor outcomes. Thus, the present synthesis provides a pragmatic estimate of the class effect of adjunctive NIVR therapy rather than the efficacy of any single device. This heterogeneity may limit the generalizability of pooled results and complicates interpretation of the overall treatment effect. Third, detailed subgroup analyses (e.g., comparisons based on intervention duration and ADL assessment tool types) offer nuanced insights into how intervention characteristics influence outcomes. Fourth, this review focused on NIVR systems, which are more accessible and cost-effective, and are typically associated with fewer side effects such as motion sickness. This highlights a practical and scalable rehabilitation approach particularly suited to home or community settings.

The conclusions of this review should be interpreted in light of the following limitations, each accompanied by an assessment of its potential impact on the pooled estimates and overall certainty of evidence.

#### Limited database coverage

The literature search was restricted to PubMed, Web of Science, and Scopus. Although these represent major biomedical and multidisciplinary databases, other subject-specific databases relevant to rehabilitation technology—such as CINAHL, PEDro, OTseeker, IEEE Xplore, Embase, and CENTRAL—as well as clinical trial registries, were not searched.

*Potential impact on pooled estimates*: the omission of these databases may have resulted in the exclusion of eligible studies, particularly those published in rehabilitation engineering and allied health literature. If missing studies systematically differed from included studies (e.g., smaller effect sizes, negative results, or different patient populations), the pooled estimates could be biased. Given that published studies tend to report larger effect sizes than unpublished trials (publication bias), the exclusion of grey literature and trial registries may have led to an overestimation of treatment effects. Therefore, the current pooled estimates should be considered potentially inflated, and the overall certainty of evidence is reduced.

#### Language restriction

This review was restricted to English-language publications. Substantial stroke rehabilitation and VR research originates from non-English-speaking regions with active NIVR programs (e.g., East Asia, Europe, Latin America). Relevant trials published in Chinese, Japanese, Korean, German, French, Spanish, or Portuguese may have been excluded.

*Potential impact on pooled estimates*: language restriction can introduce geographic and publication bias. Studies published in languages other than English may differ systematically in their effect sizes; some empirical evidence suggests that non-English trials may report smaller or null effects. Consequently, the exclusive inclusion of English-language studies may have overestimated the pooled effect sizes. This limitation also reduces the global generalizability of the findings, as the evidence base disproportionately represents English-dominant countries. The certainty of evidence is therefore lower for non-English-speaking healthcare contexts.

#### Dose confounding (unmatched therapy time)

In 3 of 12 included studies (29, 36, 40), the experimental group received additional therapy time equivalent to the VR session duration, whereas the control group did not receive a time-matched alternative. Consequently, the observed improvements in these studies may be attributable, at least in part, to increased total therapy time rather than to the specific features of NIVR.

*Potential impact on pooled estimates*: dose confounding represents a major threat to internal validity. If additional therapy time independently improves outcomes (a well-established finding in stroke rehabilitation), then the inclusion of these three studies would bias the pooled estimates away from the null, inflating the apparent effect of NIVR. In a worst-case scenario, the entire observed effect could be attributable to increased dose rather than NIVR-specific mechanisms. Although sensitivity analysis excluding these three studies (data not shown, available upon request) still showed a statistically significant pooled effect, the magnitude of the effect was substantially reduced (e.g., FMA-UE MD decreased from 5.40 to 3.91), confirming that the reported estimate is at least partially attributable to increased therapy dose. The point estimates reported in this review should therefore be understood as reflecting the combination of NIVR plus potentially greater therapy dose, rather than a pure NIVR-specific effect. This limitation substantially reduces the certainty of evidence regarding the specific efficacy of NIVR.

#### Use of RoB 1 instead of RoB 2

This review used the original RoB 1 rather than the currently recommended RoB 2 tool. This decision was made at the protocol stage and applied consistently across all included studies. However, RoB 2 offers outcome-specific assessments and a more structured framework for evaluating bias from deviations from intended interventions.

*Potential impact on pooled estimates*: the use of RoB 1 may have resulted in less nuanced bias assessments for specific outcomes. If the true risk of bias in individual studies is higher than captured by RoB 1, the pooled estimates may be overly optimistic. Conversely, if RoB 1 overestimates bias in some domains, the certainty assessment may be unnecessarily downgraded. The overall impact is difficult to quantify, but the use of an outdated tool introduces uncertainty into the risk-of-bias judgments that underpin the certainty of evidence.

#### Use of post-intervention final scores

All meta-analyses in this review were based on post-intervention final scores rather than change-from-baseline scores or ANCOVA-adjusted between-group estimates.

*Potential impact on pooled estimates*: the use of final scores assumes perfect baseline balance between groups—an assumption that may not fully hold in small-sample rehabilitation trials. If baseline imbalances existed (e.g., the NIVR group having systematically higher or lower baseline FMA-UE scores), the pooled estimates could be biased in either direction. Change scores or adjusted estimates would provide greater statistical power by accounting for baseline values; however, the included studies did not consistently report these data, precluding their use. The current pooled estimates may therefore have reduced precision and potentially biased effect sizes if baseline differences were present.

#### Non-extraction of adverse event and adherence data

This review did not systematically extract data on adverse events, cybersickness, participant adherence, dropouts attributable to intervention burden, supervision requirements, equipment costs, technical support needs, home safety considerations, or caregiver involvement.

*Potential impact on pooled estimates*: the absence of these data does not directly bias the pooled effect estimates for efficacy outcomes. However, it severely limits the assessment of the overall certainty of evidence using GRADE frameworks, which require consideration of undesirable effects. Statements in this review regarding the affordability, tolerability, and suitability of NIVR for home- and community-based settings should be regarded as hypothesis-generating rather than evidence-based conclusions. The overall certainty of evidence for implementation outcomes (as opposed to efficacy outcomes) is very low.

#### Variability in control conditions

The control conditions varied across studies. While 9 studies used conventional rehabilitation alone as the control, one study (30) used non-VR recreational activities (e.g., card games, bingo, Jenga) to match for attention and engagement, and another study (31) used group-based rehabilitation.

*Potential impact on pooled estimates*: heterogeneity in control conditions may influence the pooled effect estimates, as recreational activities or group-based rehabilitation may have different therapeutic value compared to conventional rehabilitation alone. If recreational activities provide some therapeutic benefit (e.g., through social interaction or cognitive engagement), the true effect of NIVR relative to conventional rehabilitation alone could be underestimated in that study. Conversely, if group-based rehabilitation is less effective than individualized conventional therapy, the effect of NIVR could be overestimated. The present analysis cannot disentangle the relative contributions of these different control types, introducing uncertainty into the pooled estimates.

#### Small sample sizes and lack of long-term follow-up

Many included studies had relatively small sample sizes and lacked long-term follow-up data.

*Potential impact on pooled estimates*: small sample sizes increase the risk of imprecision (wide confidence intervals) and may inflate effect size estimates due to publication bias (small-study effect). The lack of long-term follow-up means that the pooled estimates reflect only immediate post-intervention effects; the durability of observed improvements remains unknown. If effects diminish over time, the current estimates would overstate the long-term efficacy of NIVR. The certainty of evidence for sustained benefits is therefore low.

#### Clinical heterogeneity across stroke stage, intervention protocols, VR platforms, and outcome measures

While all included studies focused on upper limb rehabilitation after stroke, they differed substantially in patient characteristics (acute, subacute, or chronic stroke; varying baseline severity), intervention protocols (duration 2–12 weeks, frequency 3–5×/week, session length 20–60 min), VR platforms (gaming consoles, dedicated rehabilitation systems, motion-tracking systems), and outcome measures (FMA-UE, BBT, BI, MBI, FIM).

*Potential impact on pooled estimates*: this clinical heterogeneity complicates meta-analytic pooling and may contribute to the moderate-to-high statistical heterogeneity observed in some analyses (e.g., MBI subgroup: I^2^ = 78%). The pooled estimates should therefore be interpreted as representing a class effect of adjunctive NIVR rather than the efficacy of any single device, protocol, or patient subgroup. Meta-regression or subgroup analyses to explore sources of heterogeneity were not feasible due to the limited number of included studies.

#### Pooling different ADL instruments (BI, MBI, FIM) that assess distinct functional constructs

The BI, MBI, and FIM, while all measuring activities of daily living, assess different constructs. The BI and MBI focus on basic self-care and mobility (e.g., feeding, bathing, toileting, transfers), whereas the FIM additionally includes cognitive and social function domains (e.g., communication, social interaction, problem-solving).

*Potential impact on pooled estimates*: pooling these instruments may obscure domain-specific effects of NIVR. This likely explains our finding that the pooled BI/MBI analysis reached statistical significance (*p* = 0.04) while neither the BI nor MBI subgroup individually did so. The FIM analysis showed a more consistent association (MD = 5.99, *p* = 0.002), but this may reflect the inclusion of cognitive and social domains rather than upper limb-specific ADL improvements. Future trials should use standardized ADL instruments and report domain-specific subscores when possible.

##### Summary of limitations impact

Collectively, these limitations indicate that the pooled effect estimates should be interpreted as reflecting the combination of NIVR and potentially greater therapy dose, rather than a pure NIVR-specific effect. The certainty of evidence is substantially reduced for NIVR-specific efficacy claims due to dose confounding (3 studies) and restricted search scope, and very low for home-based implementation recommendations due to the absence of systematically extracted safety, adherence, and cost-effectiveness data. We explicitly recommend that these findings require confirmation by larger, rigorously designed, dose-matched randomized trials and more comprehensive systematic reviews (including multi-database searches, trial registries, and non-English publications). Until such evidence becomes available, clinicians and policymakers should consider NIVR as a promising but not definitively proven adjunctive intervention for upper limb rehabilitation after stroke.

## Conclusion

This systematic review and meta-analysis evaluated the association between NIVR as an adjunct to conventional rehabilitation and improvements in upper limb motor function and global functional independence measured by ADL scales in stroke patients. Given the limited search scope (PubMed, Web of Science, and Scopus only) and the inclusion of three studies with unmatched therapy time, the following findings should be interpreted as preliminary. The pooled analysis showed statistically significant improvements favoring the NIVR group. However, because 3 of 12 included studies provided additional therapy time to the NIVR group, these findings should be interpreted as reflecting the combination of NIVR and potentially greater therapy dose, not as a pure NIVR-specific effect. Dose-matched subgroup analysis (9 studies) is needed to isolate the specific contribution of NIVR. Furthermore, because the literature search was limited to PubMed, Web of Science, and Scopus, relevant RCTs indexed in other databases (e.g., CENTRAL, Embase, CINAHL, PEDro, OTseeker, IEEE Xplore) and unpublished trials from registries may have been omitted. Therefore, the present findings should be interpreted with caution and require confirmation in future systematic reviews with more comprehensive search strategies. Subgroup analyses exploring intervention duration and ADL assessment tool selection generated hypotheses for future investigation; however, these findings are exploratory and should not be interpreted as evidence of a dose–response relationship or differential efficacy across scale types.

Based on this potentially incomplete evidence, NIVR as an adjunct to conventional therapy may be associated with improved outcomes, although the contribution of increased therapy dose cannot be fully excluded. Regarding ADL outcomes specifically, the evidence is less robust. While the pooled BI/MBI analysis reached statistical significance (*p* = 0.04), neither the BI nor MBI subgroup individually did so, and the clinical relevance of the observed effect sizes remains uncertain when compared with MCID thresholds. The FIM analysis showed a more consistent association [MD = 5.99, 95% CI (2.27, 9.71)], though dose confounding in 3 of 4 FIM studies tempers this finding. It should also be noted that the ADL scales used in the included studies (BI, MBI, FIM) are global measures that assess multiple domains (e.g., mobility, transfers, cognition, lower limb function); therefore, any observed improvements in ADL scores may reflect broader functional gains rather than upper limb-specific recovery. Future trials should use standardized ADL instruments, prespecify MCID thresholds, and ensure dose matching between groups to isolate NIVR-specific effects. When implemented in dose-matched protocols, NIVR appears to confer benefits beyond conventional therapy alone and may be considered for integration into rehabilitation programs. Its potential for home- and community-based application warrants further investigation through implementation studies that systematically evaluate cost, safety, adherence, supervision requirements, and technical support needs.

## Data Availability

The original contributions of this study are detailed within the article. For further inquiries, please contact the corresponding author.
