# Supplementary material for: The effect of non-immersive virtual reality on upper limb motor function and activities of daily living in stroke patients: a systematic review and meta-analysis
Source: Front Neurol. 2026 Jun 30;17:1822985. doi: 10.3389/fneur.2026.1822985 (PMC13364612; doi:10.3389/fneur.2026.1822985)
Supplement: Supplementary file 1 [file Table_1.DOCX]

| **Authors** | **Country** | **Number** | **DOI Link** | **Mean age in years** | **Control Group** | **VR Equipment** | **Risk of Bias** | **Intervention frequency** | **Intervention duration** | **Dose matched** | **Outcome Indicator** |
| --- | --- | --- | --- | --- | --- | --- | --- | --- | --- | --- | --- |
| Cameirão et al.,2011 [29] | Spain | C = 8  T = 8 | DOI: 10.3233/rnn-2011-0599 | C = 65.22 ± 11.46  T = 58.77 ± 11.42 | Enhanced OT (object manipulation, grasping and releasing) | Spheroids Game Tasks (Strike, Grasp, Place) | Some concerns | 20 Min/daily, 3days/ week， | 12weeks | Yes | FMA-UE、  BI |
| Choi et al.,2014 [30] | South Korea | C = 10 T = 10 | DOI: 10.5535/arm.2014.38.4.485 | C = 64.70±11.3  T = 64.30±10.3 | OT | Nintendo Wii | Some concerns | 30 Min/daily, 5 days/ week | 4 week | Yes | FMA-UE、 BBT、  MBI |
| Kiper et al.,2013 [31] | Italy | C = 21 T = 23 | DOI: 10.1155/2014/752128 |  | TR | Virtual reality rehabilitation system | Some concerns | 2 h/daily, 5 days/week， | 4weeks | Yes | FMA-UE、  FIM |
| Lee et al.,2016 [40] | South Korea | C = 13 T = 13 | DOI: https://doi.org/10.1016/j.eujim.2016.08.166 | C = 69.92 ± 7.18 T = 66.46 ± 7.26 | GG | KINECT | Some concerns | 30 Min/session,3 days/week, | 8weeks | Yes | FMA-UE、BBT、  MBI |
| Leng et al.,2022 [32] | China | C = 26 T = 31 | DOI: 10.2196/33755 | C = 59.12 ± 11.62 T = 59.25 ± 10.70 | Standard rehabilitation (joint mobility, muscle strength training, neurodevelopmental therapy, electrotherapy, etc.) | Microsoft Xbox 360 Kinect | Some concerns | 60 Min/daily,5 days a week， | 3weeks | Yes | FMA-UE、  BI |
| Long et al.,2020 [33] | China | C = 27 T = 25 | DOI: 10.1186/s12984-020-00783-2 | C = 54.11 ± 14.81 T = 53.28 ± 15.30 | General rehabilitation (occupational therapy, physiotherapy, acupuncture) | Doctor Kinetic | Some concerns | 45 Min/session, 5 days/week， | 3weeks | The VR group adds an additional 45 minutes of VR training per day on top of this | FMA-UE、  MBI |
| Park et al.,2019 [34] | South Korea | C = 12 T = 12 | DOI: 10.1186/s12984-019-0595-8 | C = 51.5 ± 16.7 T = 53.5 ± 13.0 | Standard OT (AROM, hand skateboards, cup stacking, etc.) | Rapael Smart Board™ | Some concerns | 60 min/days, 5 days/week | 4weeks | Yes | FMA-UE、  MBI |
| Piron et al.,2010 [35] | Italy | C = 20 T = 27 | DOI: 10.1177/1545968310362672 | C = 62.2(9.75) T = 58.8 (8.3) | Standard Bobath therapy | Polhemus 3Space FasTrak | Some concerns | 60 Min/session,5 days /week, | 4weeks | Yes | FMA-UE、  FIM |
| Saposnik et al.,2016  [39] | Canada | C = 70 T = 71 | DOI: 10.1016/s1474-4422(16)30121-1 | - | Leisure activities (cards, bingo, Jenga, ball games) | Nintendo Wii | Some concerns | 60 Min/session,5 days/week， | 2weeks | Yes | BBT、  BI、  FIM |
| Shin et al.,2014 [36] | South Korea | C = 7  T = 9 | DOI: 10.1186/1743-0003-11-32 | C = 46.6 ± 5.8 T = 52.0 ± 11.9 | Standard OT (AROM, muscle strength training, ADL training) | RehabMaster | Some concerns | 60 Min/session,3days/week， | 2weeks | The intervention group exercised 20 minutes more per day | FMA-UE、  MBI |
| Shin et al.,2015 [37] | South Korea | C = 16  T = 16 | DOI: 10.1016/j.compbiomed.2015.03.011 | C = 54.67±13.4T = 53.37±11.8 | General Rehabilitation (Physiotherapy and Occupational Therapy) | RehabMaster | High risk | 30 Min /session,5 days/week， | 4weeks | Yes | FMA-UE |
| Yin et al.,2014 [38] | Singapore | C = 12 T = 11 | DOI: 10.1177/0269215514532851 | C = 62 T = 56 | Standard rehabilitation: physiotherapy (stretching, strength training, balance training, gait training) + occupational therapy (functional training) | Sixense electromagnetic sensors | High risk | 30Min/session,5days/week， | 2weeks | The VR group spent an average of about 1.6 hours longer | FMA-UE、  FIM |

Gaming System; FMA-UE, Fugl-Meyer Assessment Upper Extremity; BBT, Box and Block Test; BI, Barthel Index; MBI, Modified Barthel Index; FIM, Functional Independence Measure; ADL, Activities of Daily Living.

C: Control Group; D: Experimental Group; RGS: Rehabilitation Gaming System; FMA-UE: Fugl-Meyer Assessment Scale for the Upper Extremities;

OT: Stretching and muscle strength training for the full range of motion of the upper limbs; task-oriented training for activities of daily living (ADL); fine motor skills training; sensorimotor rehabilitation training;

TR: Traditional rehabilitation training supervised by a therapist on a one-to-one basis, including: various movement exercises in the horizontal and vertical planes (shoulder, elbow, forearm, hand); postural control, hand positioning, manipulation and functional skills training, and proximal-distal coordination training; examples of movements: shoulder flexion/extension/abduction/rotation, elbow flexion/extension, forearm pronation/supination, hand grasping/releasing, etc.; some movements may be performed with the therapist’s assistance;

GG: Uses the same training content as VRG (PNF movement patterns), but in a group setting; - The group training is led by a physiotherapist; there is no individualised feedback, but natural social interaction between patients (such as encouragement and praise) is permitted.
